# Delayed maturation of thymic epithelium in mice with specific deletion of β-catenin gene in FoxN1 positive cells

**DOI:** 10.1007/s00418-021-02012-w

**Published:** 2021-07-12

**Authors:** Sara Montero-Herradón, Agustín G. Zapata

**Affiliations:** 1 Department of Cell Biology, Faculty of Biology, Complutense University of Madrid, C/ José Antonio Nováis 2, 28040 Madrid, Spain; 2 Health Research Institute, Hospital 12 de Octubre (imas12), Madrid, Spain

**Keywords:** Wnt signalling, β-catenin, Thymic epithelial cells (TECs), Thymocytes

## Abstract

Wnt signalling pathways have been reported to be involved in thymus development but their precise role in the development of both thymic epithelium (TE) and thymocytes is controversial. Herein, we examined embryonic, postnatal and adult thymi of mice with a specific deletion of β-catenin gene in FoxN1^+^ thymic epithelial cells (TECs). Together with a high postnatal mouse mortality, the analysis showed severe thymic hypocellularity, largely due an important reduction in numbers of developing thymocytes, and delayed, partially blocked maturation of mutant TECs. Affected TECs included largely cortical (c) TEC subsets, such as immature MTS20^+^ TECs, Ly51^+^ cTECs and a remarkable, rare Ly51^+^MTS20^+^MHCII^hi^ cell subpopulation previously reported to contain thymic epithelial progenitor cells (TEPCs) (Ulyanchenko et al., Cell Rep 14:2819–2832, 2016). In addition, altered postnatal organization of mutant thymic medulla failed to organize a unique, central epithelial area. This delayed maturation of TE cell components correlated with low transcript production of some molecules reported to be masters for TEC maturation, such as EphB2, EphB3 and RANK. Changes in the thymic lymphoid component became particularly evident after birth, when molecules expressed by TECs and involved in early T-cell maturation, such as CCL25, CXCL12 and Dll4, exhibited minimal values. This represented a partial blockade of the progression of DN to DP cells and reduced proportions of this last thymocyte subset. At 1 month, in correlation with a significant increase in transcript production, the DP cell percentage increased in correlation with a significant fall in the number of mature TCRαβ^hi^ thymocytes and peripheral T lymphocytes.

**Supplementary Information:**

The online version contains supplementary material available at 10.1007/s00418-021-02012-w.

## Introduction

Wnt-mediated signals control many biological processes including cell proliferation, fate specification, cellular polarity and migration (van Loosdregt et al. 2013). Wnt signalling pathways comprises a canonical pathway, which is mediated by β-catenin and the transcription factor TCF (T-cell factor), and non-canonical pathways, which include the intracellular Wnt signalling and the planar polarity pathway (Korswagen 2002; Liang et al. 2007).

Wnt ligands (19 in humans) activate the canonical pathway by binding to surface Wnt receptors (10 of the family Frizzled -Fzd-) that form a complex with the Lrp (Low density lipoprotein receptor related protein) 5 and 6 co-receptors (Staal et al. 2008). The ligand-receptor interaction produces signals that engage APC (Adenomatous Polyposis coli protein Complex), eventually inhibiting the catalytic activity of glycogen synthase kinase 3β, which results in stabilization of β-catenin. Stable β-catenin moves into the nucleus where it forms a complex with TCF/LEF-1 (Lymphoid Enhancer Factor 1) leading to activation of Wnt target genes, such as FoxN1, BclxL, Axin, cyclin D1 and c-myc (Miller 2002). Thus, β-catenin levels directly correlate with the activity of canonical Wnt signalling (Staal and Langerak 2008; Clevers and Nusse 2012).

A role for Wnt signalling pathways in thymus biology was first suggested by the expression of distinct components of the route in different thymic cell subsets (Mulroy et al. 2002; Pongracz et al. 2003). Both thymocytes and especially thymic epithelial cells (TECs), express Wnt ligands and Fzd receptors, but the expression varies in distinct cell subsets and during thymic development. Cortical (c) TECs show high levels of Wnt4, Wnt7b, Wnt9a and Wnt10a, whereas immature MHCII^lo^ medullary (m) TECs express Wnt4 and Wnt10a, and mature MHCII^hi^ ones express Wnt10a and Wnt10b (Brunk et al. 2015). In addition, Wnt4 is particularly abundant in the immature MTS24^+^ TEC (Heinonen et al. 2011), whereas Wnt1 is expressed in both MTS24^+^ and MTS24^−^ cells (Balciunaite et al. 2002). On the other hand, TECs express the Wnt receptors Fzd2, Fzd4, Fzd5-8 and Fzd10 (Pongracz et al. 2003). In thymocytes, Wnt4 is expressed principally in DP and SP (both CD4^+^ and CD8^+^) cells, whereas Wnt5b transcripts reach maximal levels in DP cells, sharply decreasing in SP thymocytes. The expression of both molecules is significantly lower in peripheral T cells (Balciunaite et al. 2002). Fzd6 receptors predominate in DN cells, drastically decreasing in DP thymocytes, whereas, on the contrary, Fzd5 is not expressed in this thymic cell subset and becomes predominant in DP thymocytes (Pongracz et al. 2003). Other components of the Wnt signalling pathway such as Dvl1 (Dishevelled 1), TCF, LEF-1 and Kremen, an inhibitor of the canonical Wnt pathway (Osada et al. 2006), have been demonstrated to be expressed in both freshly isolated mature primary TECs and cortical and medullary epithelial cell lines (Balciunaite et al. 2002).

Numerous studies have addressed the role of Wnt signalling in T cell differentiation, claiming that it is necessary for a proper functional T-cell differentiation, including T-cell specification, positive/negative selection and phenotypic thymocyte maturation (Staal and Clevers 2003; Ma et al. 2012; Brunk et al. 2015). However, information on its involvement in the development and organization of thymic epithelium is less conclusive and sometimes contradictory***.*** It has been pointed out that β-catenin-mediated canonical Wnt signalling pathway is required for the development (Zuklys et al. 2009; Liang et al. 2013), homeostasis (Liang et al. 2013) and regeneration (Bredenkamp et al. 2014) of TECs, but recently, thymic development was reported to only occur when β-catenin-dependent Wnt signalling is low or absent (Swann et al. 2017). Other results support the abrogation of canonical Wnt signalling corresponds to deficits in the maturation of thymic epithelium (Osada et al. 2010; Heinonen et al. 2011; Kvell et al. 2014; Swann et al. 2017; Tan and Nusse 2017), whereas constitutive activation of the Wnt pathway results in altered TEC fate with terminal differentiation of epithelium (Kuraguchi et al. 2006; Osada et al. 2006; Zuklys et al. 2009; Swann et al. 2017) and/or formation of thymomas (Liang et al. 2015). Furthermore, although in some studies altered TE maturation occurs concomitantly with changes in T-cell differentiation (Kuraguchi et al. 2006; Zuklys et al. 2009; Heinonen et al. 2011; Liang et al. 2013; Swann et al. 2017), in others epithelial changes do not correspond to altered T-cell development (Osada et al. 2006, 2010).

In fact, it is difficult to compare reported results because: (1) experimental approaches are different, abrogating or overactivating distinct members of the Wnt signalling pathway; (2) described mutant mice can exhibit gene deficits in all thymic components or specifically restricted to either thymocytes or TECs; (3) even in this latter condition, promoters governing mutant gene expression can be specific to all TECs (i.e., FoxN1), or just to a TEC subset (i.e., K14, K5) and its progeny; (4) finally, the parameters analysed or the stages studied (embryonic, postnatal, adult) are frequently different.

To re-evaluate information on the role played by β-catenin-dependent Wnt signalling, we examined β-catenin-deficient mice in which the molecules had been eliminated from all FoxN1^+^ TECs. Similar mutants have been described recently (Swann et al. 2017), but the study was limited to the embryonic period because mutants died just after birth from functional skin problems, since FoxN1 is also expressed by the epidermal basal cells. Although in our hands survival of the obtained mutants was also very low (< 50%) because of epidermic alterations, we characterized the phenotype of embryonic, postnatal and surviving adult mutant mice, thus obtaining new information on the role played by β-catenin-dependent Wnt pathway in the TEC maturation. Remarkably, mice with specific deletion of Grp177, a molecule necessary for Wnt ligand secretion, in FoxN1^+^ TECs are viable, although exhibit mild hair loss and thymic hypotrophy that affects both thymocytes and TECs (Brunk et al. 2015).

Our results confirm a role for β-catenin-dependent canonical Wnt signalling pathway in the thymic epithelium maturation, largely affecting immature MTS20^+^ cells, some of which are presumptive bipotent TEPCs. In turn, partial blockage of TEC development affects T-cell differentiation and positive selection of thymocytes. Our study also identifies some molecules, master regulators for TEC development, particularly affected by the absence of β-catenin.

## Materials and methods

### Mice

We crossed mice with loxP-flanked alleles encoding β-catenin beta-1 (Ctnnb1^fl/fl^; B6.129-Ctnnb1^tm2Kem^/KnwJ) (Brault et al. 2001) with FoxN1Cre-transgenic mice [FoxN1^Cre^; B6(Cg)-FoxN1^tm3(cre)Nrm^/J] (Gordon et al. 2007) to generate knockout mice with the β-catenin beta1 gene specifically deleted in FoxN1^+^ TECs. Both mice were provided by The Jackson Laboratory (Bar Harbor, ME, USA). In all assays, FoxN1^Cre^ was considered as wild-type (WT) mice, and mutant mice as Ctnnb1cKO (FoxN1^Cre^; Ctnnb1^fl/fl^). At least five animals (WT and mutants) were used for each experimental group.

All animals were bred and maintained under pathogen-free conditions in the animal care facilities of the Complutense University of Madrid. The day of vaginal plug detection was designated as day 0.5 for determining the age of foetuses.

### Detection of recombined alleles

Genotyping was performed on tail biopsies using conventional PCR. Primers specific for the genotyping were used as described by Brault et al. (2001). The primers used for detecting FoxN1^Cre^ and β-catenin beta-1 wild-type, floxed and recombinant alleles are included in the Supplementary Material (Online Resource 1).

### Animal statement

The study and all experimental procedures were carried out in accordance with the recommendations of the “Ethics Committee for Animal Research” of the Complutense University of Madrid. The protocols were approved by the Regional Government of Madrid.

### Cell suspensions and flow cytometry analysis

#### Foetal total thymic cell suspensions

E13.5, E15.5 or E17.5 thymic lobes were isolated and disaggregated using Liberase TM (Roche) at 1 U/mL together with DNAse I at 0.1 mg/mL (Roche) for 15 min at 37 °C in a water-bath and later gently pipetted to obtain single-cell suspensions.

#### Enriched TEC suspensions from postnatal and adult thymi

Postnatal (P15) and 1-month-old (1 M) thymi were removed from the thoracic cavity and cut with foetal scissors, and thymocytes were depleted by gently pipetting with a wide-bore glass pipette in cold RPMI 1640. Thymic fragments were poured twice; the supernatants, mainly containing thymocytes, were removed and thymic fragments disaggregated, as described above.

#### Thymocyte suspensions

Thymi, spleens and inguinal lymph nodes (ILNs) were mechanically fragmented using a homogenizer with RPMI medium at 2% foetal bovine serum (FBS) and then filtered to remove cellular debris. For the spleen and peripheral blood, the red blood cells were lysed with lysing solution (160 mM NH_4_Cl, 5.7 mM K_2_HPO_4_, and 0.1 mM EDTA) for 10 min on ice.

All cell suspensions obtained were washed, resuspended in FACS buffer (PBS1x + 1%FBS + 10 mM EDTA) and stained for 15 min at 4 °C with specific fluorescence monoclonal antibodies (mAbs) (see Online Resource 2). After first staining, cells were washed in PBS and for detecting UEA1 (*Ulex Europaeus* Agglutinin Lectin 1)-Biotin (Vector Laboratories) and MTS20 supernatant (kindly provided by Dr Ann Chidgey) incubated with secondary antibody Streptavidin-PERCP (BD Biosciences) and goat anti-rat IgM-Alexa Fluor488 (ThermoFisher Scientific) or goat anti-rat IgM-PE (Jackson ImmunoResearch), respectively, for 15 min at 4 °C in PBS. For caspase-3 and β-catenin expression, cell suspensions were fixed and permeabilised using fixation/permeabilization solution according to the manufacturer’s instructions (BD Biosciences) and incubated with the corresponding antibodies at room temperature (RT) for 40 min in dark or at 4 °C for 30 min in dark, respectively. Before analysis, stained cell suspensions were washed in PBS, resuspended in FACS buffer and analysed in a FACS Calibur (BD Biosciences) or FACSAriaIII (BD Biosciences) from the Centre for Cytometry and Fluorescence Microscopy of the Complutense University of Madrid. In all cases, non-viable cells were excluded by forward-side scatter and the analyses were performed with FCS Express III software (DeNovo Software, Los Angeles, CA, USA).

### Apoptosis assays

Total thymic cells from either WT or β-catenin-deficient mice were washed in Annexin buffer (BD Pharmigen, BD Biosciences) and incubated with AnnexinV-Brilliant Violet 605 (BD Horizon, BD Biosciences) plus anti-EpCAM, anti-CD45 and anti-Ly51 mAbs, MTS20 supernatant and UEA1-Biotin for 20 min at RT in the dark. Cells stained with MTS20 supernatant and UEA1-Biotin were incubated with secondary antibodies and then resuspended in Annexin buffer. Five to ten minutes before analysis, Sytox Blue Dead Cell Stain (ThermoFisher Scientific) was added. Apoptotic cells were identified as AnnexinV^+^/Sytox Blue^−^ cells and analysed in a FACSAriaIII cytometer (BD Biosciences) at the Cytometry and Fluorescence Microscopy Centre of the Complutense University of Madrid.

### Cell cycle analysis

Both WT and mutant thymic cells obtained as previously described were incubated with anti-EpCAM, anti-CD45, anti-Ly51 mAbs, MTS20 supernatant and UEA1-Biotin. After incubation with secondary antibodies, cells were washed in PBS and incubated in Cytofix/Cytoperm solution (BD Biosciences) at 4 °C for 30 min in dark. Then, cells were washed twice with Perm/Wash buffer (BD Biosciences) and resuspended in a solution of Perm/Wash containing 5 μg/mL of Hoechst 33342 (ThermoFisher Scientific) at 4 °C for 1 h and 30 min in dark. Cells were analysed in a FACSAriaIII cytometer (BD Biosciences) at the Cytometry and Fluorescence Microscopy Centre of the Complutense University of Madrid. Cycling cells corresponded to cells in S + G_2_/M cell cycle phase.

### Immunofluorescence and semi-quantification analysis

Cryosections were obtained from both E13.5 and E15.5 (12 μm thick) and E17.5, P15 and 1 M (10 μm thick) WT and Ctnnb1cKO thymi, fixed in acetone at RT for 10 min and air dried. Cryosections were stained with primary antibodies specific for K5 (Rabbit, polyclonal; Covance), K8 (Rat, clone Troma-1; Developmental Studies Hybridoma Bank), AIRE (Rat, clone 5H12, BD Bioscience), MTS20 and MTS10 supernatants (Rat IgM; kindly donated by Dr Ann Chidgey from Monash University) for 1 h at RT. After washing three times in cold PBS for 5 min, sections were incubated with the following secondary antibodies: goat anti-rat IgM-Dylight594 (Jackson ImmunoResearch), donkey anti-rabbit IgG-AlexaFluor488 or AlexaFluor647, donkey anti-rat IgG-AlexaFluor594 or AlexaFluor488 or chicken anti-rat IgG-AlexaFluor647 (ThermoFisher Scientific) for 45 min at RT. Sections were then washed three times in cold PBS for 5 min and mounted with antifade Prolong Gold (ThermoFisher Scientific). Image acquisition was performed using a Leica SP8 confocal microscope equipped with a HC PL APO CS2 20×/0.75 DRY objective and LasX software from the Cytometry and Fluorescence Microscopy Centre of the Complutense University of Madrid.

In both WT and mutant thymi, the number of AIRE^+^ cells was counted and related to the MTS10^+^ medullary thymic area in mm^2^ in a minimum of ten non-overlapping medullary islets of at least three different thymi. All semi-quantitative analyses were carried out using Image J software.

### RNA isolation, RT-PCR and real-time PCR (qPCR)

EpCAM^+^CD45^−^ TECs were sorted from either P15 and 1 M WT or Ctnnb1cKO thymi using a FACSAriaIII cell sorter (BD Biosciences) at the Cytometry and Fluorescence Microscopy Centre of the Complutense University of Madrid. Total RNA from isolated TECs was obtained using an RNAqueous^®^-Micro Kit (ThermoFisher Scientific) according to the manufacturer’s instructions. cDNA synthesis was performed by RT-PCR with the High-Capacity cDNA *Reverse* Transcription kit (ThermoFisher Scientific, USA), using 0.1 µg of RNA according to the manufacturer’s instructions. The expression of different genes was determined by real-time PCR (qPCR) using TaqMan Gene expression assays. The HPRT gene was used as a housekeeping gene. All primers used (see Online Resource 3) were purchased from ThermoFisher Scientific.

Efficiency of the amplification reaction and Ct values for each gene were obtained using a QuantStudio 12 K FLEX PCR system with QuantStudio Real-Time PCR Software v1.2.2. at the Genomic Centre of the Complutense University of Madrid. The relative expression of each sample was normalized to HPRT1 values and represented as RQ (2^−ΔΔCt^). Data show the mean of the three independent experiments.

### Statistical analysis

For phenotypical analysis, data were expressed as mean ± SD from at least five independent experiments. The significance of differences with respect to control values was analysed by the Student’s *t *test after analysis of *f *test data. The software Microsoft Excel 365 (Redmond, WA, USA) and GraphPad Prism 8 (San Diego, California) were used for statistical procedures and graph creation, respectively. The significance of the *p* value obtained comparing different developmental stages or between WT and mutant mice was indicated as *p ≤ 0.05, **p ≤ 0.01, and ***p ≤ 0.001.

## Results

**Fig. 1:**
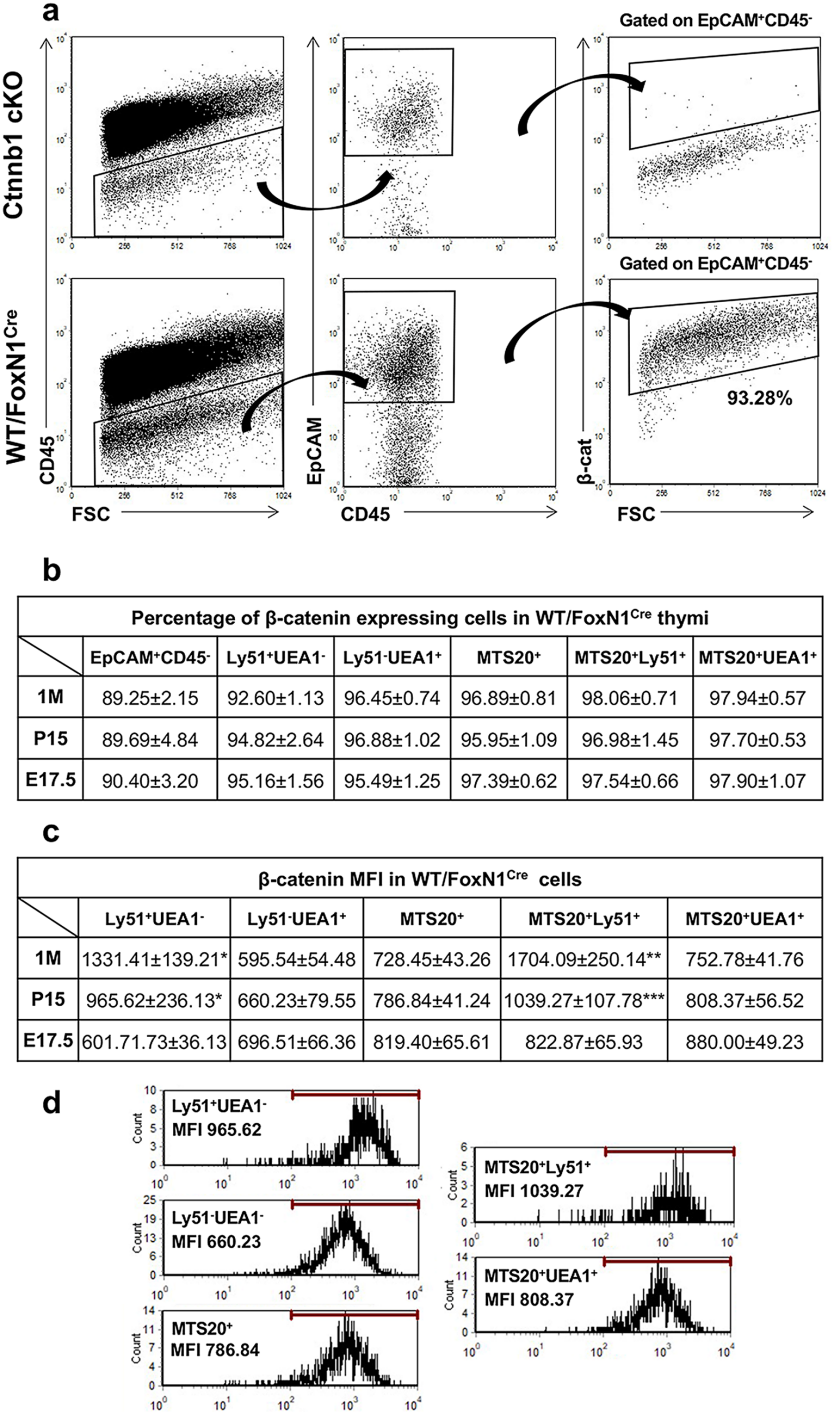
Expression of β-catenin in WT/FoxN1^Cre^ and Ctnnb1 cKO thymi. **a** Dot plots show a representative example of the absence of β-catenin expression in the EpCAM^+^CD45^−^ TECs of P15 Ctnnb1cKO mice with respect to WT ones. **b** Percentages of β-catenin-positive cells in total WT/FoxN1^Cre^ EpCAM^+^CD45^−^ TECs, Ly51^+^UEA1^−^ cTECs, Ly51^−^UEA1^+^ mTECs, immature MTS20^+^ cells, MTS20^+^Ly51^+^ cTECs and MTS20^+^UEA1^+^ mTECs. In all studied TEC subsets, the proportions of β-catenin expressing TECs are high, particularly in the most immature MTS20^+^ cortical and medullary TEC subsets. **c** β-catenin expression by cell (MFI) in distinct control, FoxN1^Cre^ TEC subsets. Note the increased expression of β-catenin after birth in the Ly51^+^UEA1^−^ cTEC compartment, particularly within the MTS20^+^ cTEC subset. Contrarily, β-catenin expression in mTECs is not significantly affected. **d** Representative examples of the MFI expression in different TEC subsets. The significance of the Student’s *t* test probability between the studied stages (1 M vs. P15; P15 vs. E17.5) is indicated as: *p ≤ 0.05, **p ≤ 0.01 and ***p ≤ 0.001

We first examined the conditions of embryonic, postnatal (P15) and adult (1-month-old, 1 M) thymic epithelium of mice with selectively deleted β-catenin gene in FoxN1^+^ cells, which did not express β-catenin (Fig. 1a). Then, we studied thymocyte differentiation in these mutants and the possible underlying mechanisms involved in these processes.

### β-Catenin expression in TECs of WT mice

Because the amount of β-catenin present in a given cell type has been found to reflect signalling pathway activity (Staal and Langerak 2008; Clevers and Nusse 2012), we evaluated its expression by distinct TEC populations at different stages of WT thymus development (Fig. 1b, c, Online Resource 4). At all the stages analysed, most total EpCAM^+^CD45^−^ TECs (around 90%) expressed β-catenin, particularly the immature MTS20 cell population, although no significant differences occurred between the proportions of β-catenin positive cells when the Ly51^+^UEA1^−^ and Ly51^−^UEA1^+^ cell subsets were compared (Fig. 1b). However, the levels of β-catenin expression by cells were significantly different between TEC subsets (Fig. 1d) and when different stages of development were compared within the same TEC subpopulation (Fig. 1c). At E17.5, β-catenin expression by cells, measured as MFI, was similar in total Ly51^+^UEA1^−^ TECs and Ly51^−^UEA1^+^ TECs, but within these populations the highest expression corresponded to immature MTS20^+^Ly51^+^ and MTS20^+^UEA1^+^ cell populations (Fig. 1c). At postnatal stages, while this expression did not change at P15 and 1 M adult thymi in either Ly51^−^UEA1^+^ or MTS20^+^UEA1^+^ cells, in the Ly51^+^UEA1^−^ cells, and even more in the MTS20^+^Ly51^+^ cell population, it increased gradually and significantly throughout development (Fig. 1c).

### Thymic cell content and maturation of TEC subsets

**Fig. 2:**
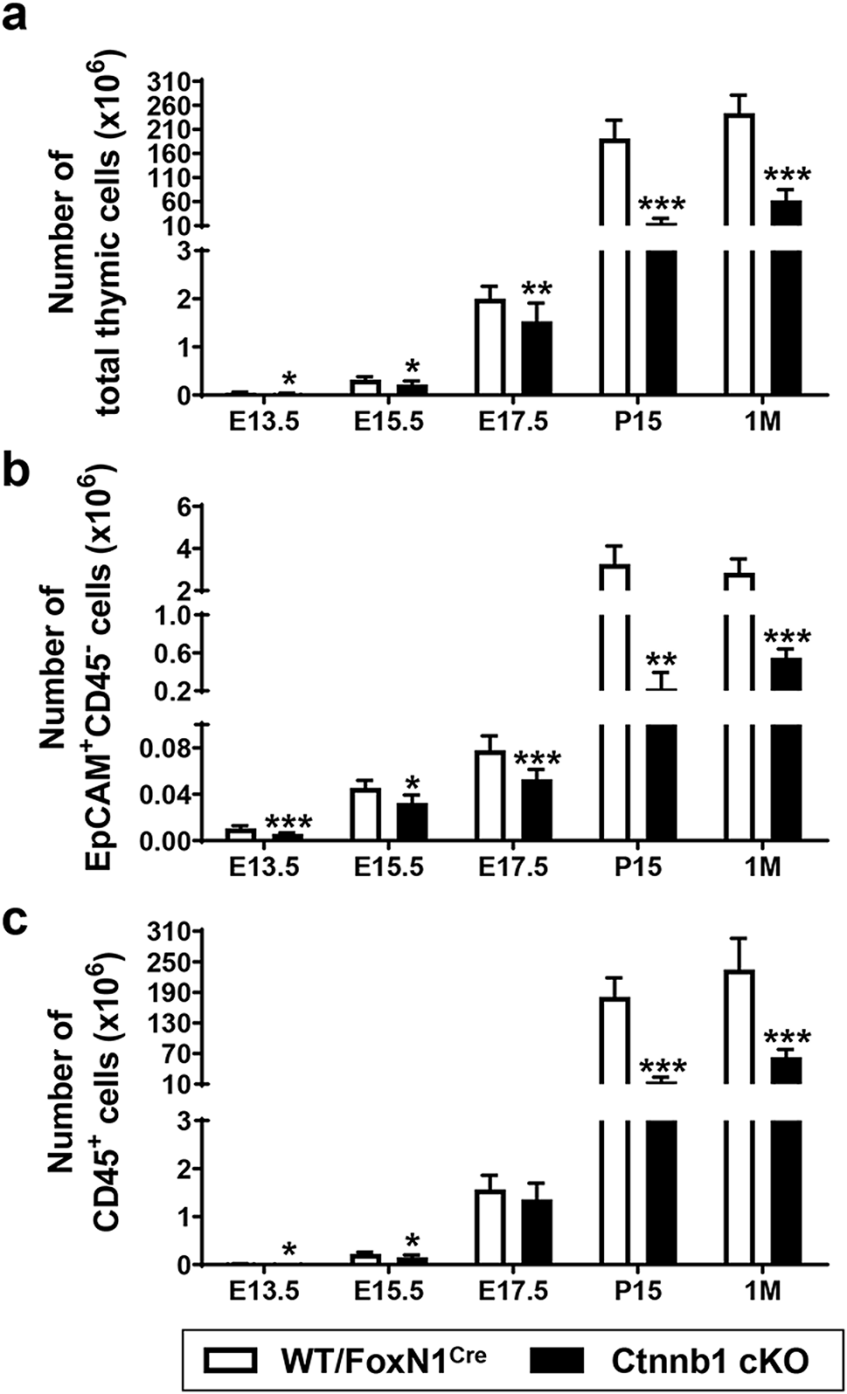
Decreased cellularity in Ctnnb1 cKO thymi. Comparative analysis of the numbers of total thymic cells (**a**), EpCAM^+^CD45^−^ TECs (**b**) and CD45^+^ cells (**c**) in both WT/FoxN1^Cre^ and Ctnnb1 cKO thymi at different stages of thymus development. Note that the reduced cell content affects both the CD45^+^ lymphoid cell populations and the TECs. The significance of the Student’s *t* test probability between WT/FoxN1^Cre^ and mutant mice is indicated as: *p ≤ 0.05, **p ≤ 0.01 and ***p ≤ 0.001

In all stages studied, the total number of thymic cells was significantly lower in mutant thymi than in WT ones, with differences that gradually increased throughout development (Fig. 2a), affecting both EpCAM^+^CD45^−^ TECs (Fig. 2b) and CD45^+^ thymocytes (Fig. 2c). However, in absolute terms, the contribution of thymocytes was higher than that of TECs.

**Fig. 3:**
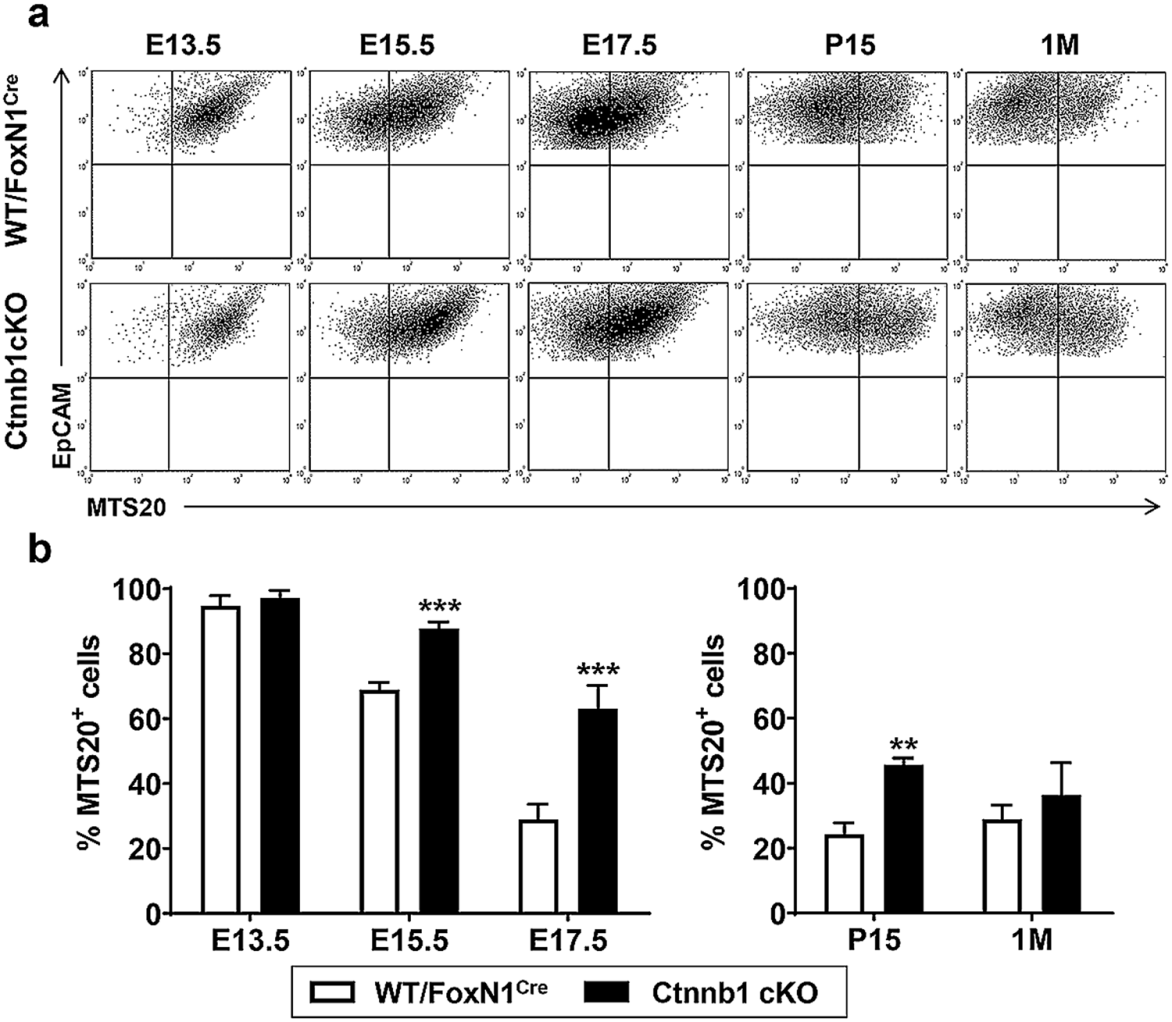
Progression of the immature MTS20^+^ TECs through thymus development. **a** The panel shows a representative dot plot of the MTS20^+^ cell population in the EpCAM^+^CD45^−^ TEC compartment of either WT/FoxN1^Cre^ or Ctnnb1 cKO mice. **b** The proportions of immature MTS20^+^ cells accumulate during the development of Ctnnb1 cKO mice, showing significantly higher values than those found in WT/FoxN1^Cre^ thymi. The significance of the Student’s *t* test probability between WT and mutant is indicated as: **p ≤ 0.01 and ***p ≤ 0.001

**Fig. 4:**
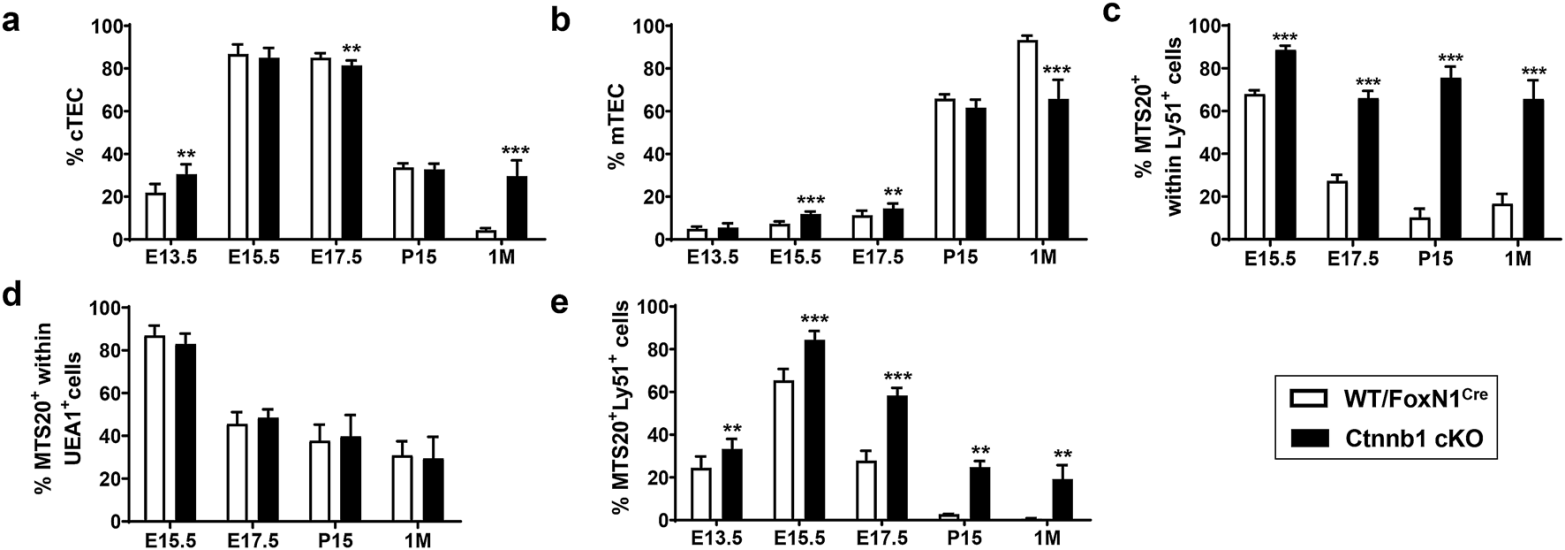
Effects of the Ctnnb1 gene deletion in FoxN1^+^ cells on the maturation of cortical and medullary TEC subsets. Different TEC subpopulations, gated in the EpCAM^+^CD45^−^ cell compartment, were defined by the expression of Ly51/UEA1/MTS20 through thymus development in both WT/FoxN1^Cre^ and Ctnnb1 cKO mice. Frequency of Ly51^+^UEA1^−^ cTECs (**a**) and Ly51^−^UEA1^+^ mTECs cells (**b**). Proportions of MTS20^+^ within either Ly51^+^ cTECs (**c**) or UEA1^+^ mTECs (**d**). Total proportions of MTS20^+^Ly51^+^ cTEC (**e**) in mutant and WT thymi. The significance of the Student’s *t* test probability between WT and mutant is indicated as: **p ≤ 0.01 and ***p ≤ 0.001

**Fig. 5:**
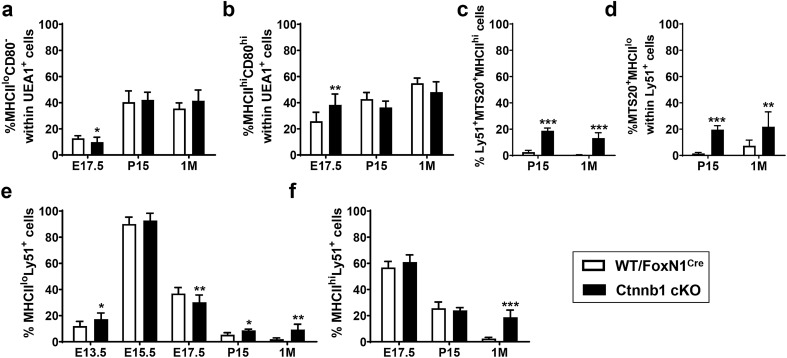
Proportions of mature and immature TECs defined by MHCII expression in WT/FoxN1^Cre^ and Ctnnb1 cKO mice. Proportions of immature MHCII^lo^CD80^−^ **(a)** and mature MHCII^hi^CD80^hi^ cells (**b**) within the UEA1^+^ mTEC subset. Total proportions of presumptive Ly51^+^MTS20^+^MHCII^hi^ thymic epithelial cell progenitors (**c**). Proportions of MTS20^+^MHCII^lo^ cells within the Ly51^+^ cTEC compartment (**d**). Proportions of MHCII^lo^Ly51^+^ (**e**) and MHCII^hi^Ly51^+^ cells (**f**) in both WT/FoxN1^Cre^ and Ctnnb1 cKO mice. The significance of the Student’s *t* test probability between WT and mutant is indicated as: *p ≤ 0.05, **p ≤ 0.01 and ***p ≤ 0.001

Analysis of the progression of distinct epithelial cell subpopulations showed that, in general, this was altered in mutant thymi compared to WT ones, resulting in a significant accumulation of the proportions of some TEC subsets, suggesting a partial blockade of their maturation (Fig. 3–5).

MTS20 is a marker of immature TECs, first described as a presumptive TEPC (Depreter et al. 2008), whose expression predominates in the earliest stages of development to gradually decrease later on (Montero-Herradon et al. 2016). In all analysed stages, its pattern of development was similar in WT and Ctnnb1 cKO thymi (Fig. 3a), but the proportion of MTS20^+^ cells was higher in mutants than in WT thymi with significant differences from E15.5 to P15 (Fig. 3b). Even at the E13.5 stage, in which almost all cells were MTS20^+^ in both control and mutant thymi, the latter ones showed higher MTS20 expression (Fig. 3a), indicating that epithelial cell differentiation could be compromised in β-catenin-deficient mice.

Then, we analysed how this accumulation of immature TECs affected development of the cortex and medulla (Online Resource 5a). Within the EpCAM^+^ TEC population, the Ly51^+^ cTEC accumulated at E13.5 then decreased (Fig. 4a) when proportions of MTS20^+^ cells increased (Fig. 3b). After birth, when the proportions of cTECs decreased rapidly in WT thymi, in mutant mice these remained high, particularly in 1 M adult ones (Fig. 4a). Throughout embryonic development, the proportions of medullary (m) mTECs (UEA1^+^ cells) accumulated in mutant thymi (Fig. 4b) but, after birth, when there was an important increase in medullary areas in WT thymi (Irla et al. 2013; Dumont-Lagace et al. 2014), the Ctnnb1 cKO thymi showed significantly lower proportions of mTECs than WT ones (Fig. 4b).

To analyse the contribution of immature MTS20^+^ cells to this TE phenotype, we examined the proportions of MTS20^+^ cells within the cortical (Fig. 4c) and medullary epithelial cell populations (Fig. 4d). While proportions of MTS20^+^ cells within the Ly51^+^ cTEC subset were significantly higher in mutant than in WT thymi (Fig. 4c), there were no differences within the UEA1^+^ mTEC compartment (Fig. 4d). Thus, the contribution of the MTS20^+^UEA1^+^ cell subpopulation to an altered mTEC maturation was minimal (Fig. 4d), whereas MTS20^+^Ly51^+^ cTECs seemed to accumulate in the Ctnnb1cKO thymi (Fig. 4c), as evidenced when the total proportions of MTS20^+^Ly51^+^ cells were evaluated in the mutant thymus (Fig. 4e).

These results were confirmed by studying the changes in TEC subpopulations identified by the expression of other TEC markers. The maturation of mTECs is defined by the expression of both MHCII molecules and CD80 co-receptors (Gray et al. 2007; Rossi et al. 2007) (Online Resource 5b). Thus, immature Ly51^−^UEA1^+^MHCII^−/lo^CD80^−^ mTECs are capable of giving rise to mature Ly51^−^UEA1^+^MHCII^hi^CD80^hi^ cells. Analysis of the expression of both MHCII and CD80 within the Ly51^−^UEA1^+^ mTEC population showed a slight reduction in the proportions of immature MHCII^lo^CD80^−^ mTECs (Fig. 5a) and increased values of the MHCII^hi^CD80^hi^ mature ones (Fig. 5b) in E17.5 thymi, but no differences were found after birth (Fig. 5a, b). These results confirmed the low relevance of a lack of β-catenin on mTEC populations after birth. Therefore, the lower proportions of Ly51^−^UEA1^+^ cells observed in adult mutant thymus, as compared to the WT one, would only be a consequence of the accumulation of Ly51^+^UEA1^−^ cTECs.

However, the scenario is very different in the thymic cortex of Ctnnb1cKO mice. First, it is important to remark that within the MTS20^+^Ly51^+^ cell subpopulation, UEA1^−^Ly51^+^MTS20^+^MHCII^hi^ cells have been described to contain long-term cortex-medulla common precursor cells, whereas the UEA1^−^Ly51^+^MTS20^+^MHCII^−/lo^ cell subset presents predominantly cortical-restricted precursor capability (Ulyanchenko et al. 2016). Remarkably, at both P15 and 1 M the proportions of this Ly51^+^MTS20^+^MHCII^hi^ cell subset were minimal in WT thymi but in mutant ones showed significantly higher values (Fig. 5c), reflecting a build-up of this cell population of presumptive bipotent TEC progenitors in mutant thymi. Also, the MTS20^+^MHCII^lo^ cell subset had accumulated in mutant thymi within Ly51^+^ cTECs (Fig. 5d).

On the other hand, as for the mTEC lineage, the expression of MHCII molecules defines the maturation of cTECs (Shakib et al. 2009). In WT thymi, the proportions of both MHCII^lo^ (Fig. 5e) and MHCII^hi^ cTECs (Fig. 5f) decreased gradually from E15.5 onward, whereas in the mutant ones the progression of both cell subsets was similar, but their reduction was less drastic (Fig. 5e, f). Accordingly, mutant cell subsets accumulated significantly, especially after birth (Fig. 5e, f), because they did not decrease in the thymic cortex so they did not increase in the medulla either, by contrast to observations in WT thymi.

### Immunohistochemical analysis of β-catenin-deficient and WT thymi

**Fig. 6:**
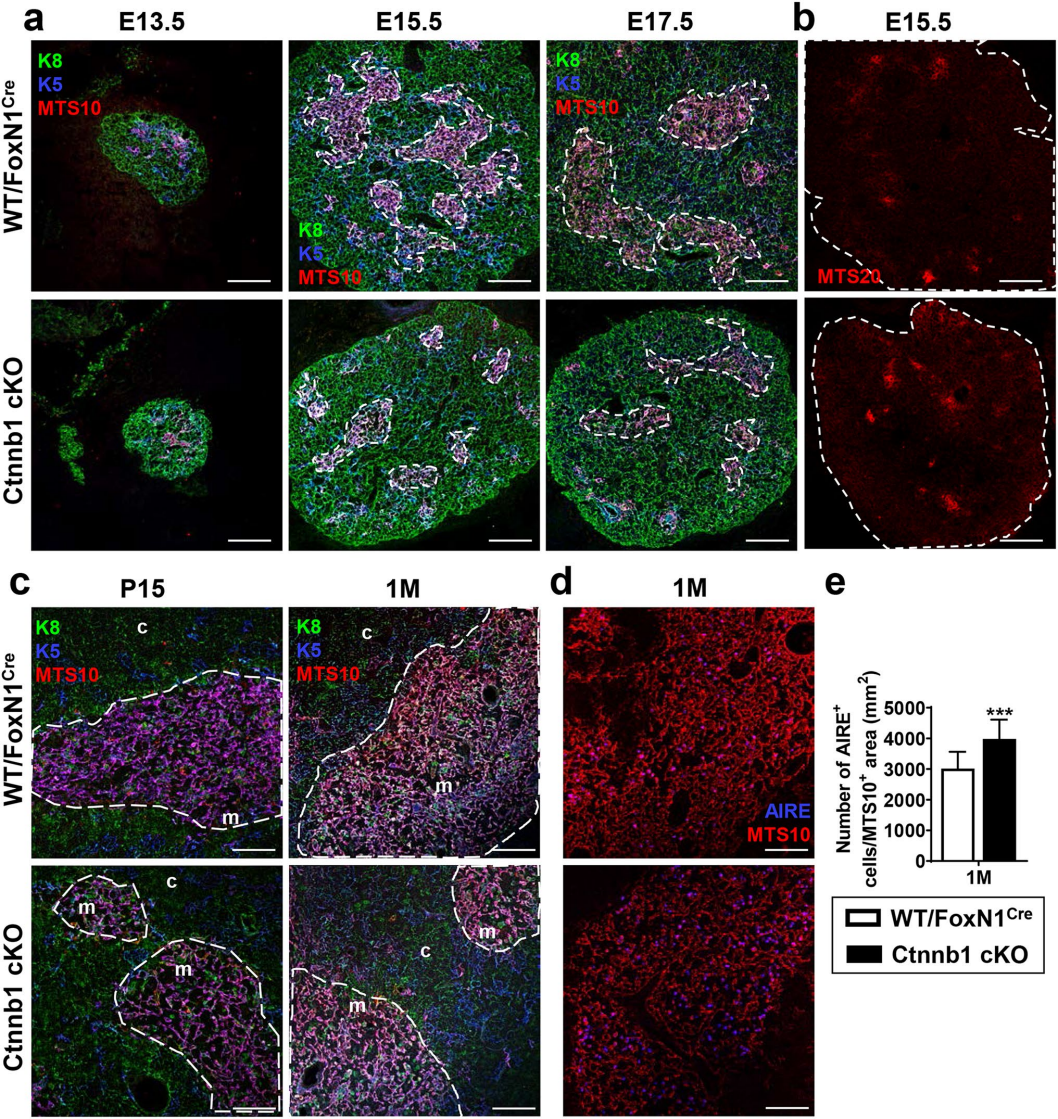
Immunofluorescence study of the maturation of thymic epithelium in WT/FoxN1^Cre^ and Ctnnb1 cKO mice. Both embryonic (**a**) and postnatal/adult (**c**) WT and mutant thymic cryosections were stained with anti-K8 (green) (cTECs), anti-K5 (blue) (mTECs) and MTS10 (red) (mTECs) mAbs to identify the distinct TEC subsets. Note the size difference of the WT and mutant thymic sections as well as the fragmented medullary foci (delimited by dotted lines), particularly in the adult mutant thymi (**c**). Mutant thymic sections also contain higher numbers of both MTS20^+^ cells (**b**) and AIRE-expressing mTECs (**d**, blue) than WT ones. In 1 M thymi, a semiquantitative study (**e**) confirmed the significant increase in the number of AIRE^+^ mTECs in the mutant thymi. The significance of the Student’s *t* test probability between WT and mutant is indicated as: ***p ≤ 0.001. Thymic cortex (**c**), thymic medulla (**m**). Scale: 100 μm

An immunohistochemical study performed on cryosections of WT and β-catenin-deficient thymi showed the same changes as the cytometric analysis confirming the delayed maturation of thymic epithelium in mutant mice. The reduced numbers of total thymic cells observed in the mutant thymi correlated well with the smaller size of mutant thymic sections as compared with WT ones (Fig. 6). In addition, the MTS10^+^ thymic medulla appeared highly fragmented, as small and scattered medullary foci without the presence of a central, unique medullary area as observed in adult WT thymi (Fig. 6c). The study also confirmed the higher numbers of immature MTS20^+^ TECs in mutant thymi, largely in the embryonic ones (Fig. 6b), and the gradual, but small increase in AIRE-expressing mTECs (Fig. 6d) in mutant thymi, a finding confirmed by semiquantitative histochemical (Fig. 6e) and qPCR studies (Fig. 10).

### Survival and proliferation of TECs

**Fig. 7:**
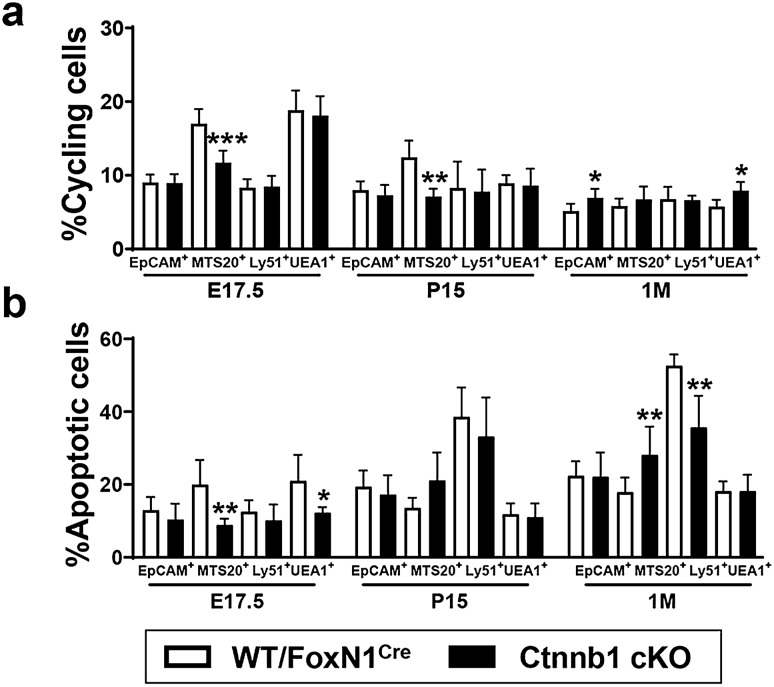
Percentages of cycling (a) and apoptotic (b) TECs in WT/FoxN1^Cre^ and Ctnnb1 cKO thymi. Total EpCAM^+^CD45^−^ TECs (EpCAM^+^), immature MTS20^+^cells, Ly51^+^UEA1^−^ (Ly51^+^) cTECs and Ly51^−^UEA1^+^ (UEA1^+^) mTECs were studied at E17.5, P15 and 1 M. In both studies, changes largely affected the immature MTS20^+^ cell population. The significance of the Student’s *t* test probability between WT and mutant is indicated as: *p ≤ 0.05, **p ≤ 0.01 and ***p ≤ 0.001

To know whether the lower content of total EpCAM^+^ TECs or the different proportions of TEC subpopulations observed in mutant thymi, as compared with the WT ones, were related to differences in cell proliferation and/or death, we examined the proportions of cycling and apoptotic cells in different TEC subsets at distinct stages of development. At E17.5 and P15, there were no significant differences in the proportions of cycling total EpCAM^+^ cells, total cTECs (Ly51^+^) or mTECs (UEA1^+^), but immature MTS20^+^ TECs underwent a significant decrease (Fig. 7a). Therefore, the described accumulation of distinct TEC subsets cannot be explained by a higher proliferation rate alone. In 1 M adult mice, the values of cycling EpCAM^+^ TECs were significantly higher in β-catenin-deficient thymi, in correlation with a higher percentage of cycling medullary UEA1^+^ TECs (Fig. 7a). On the contrary, there were no significant differences in the proportions of apoptotic mutant EpCAM^+^ cells at any of the ages studied (Fig. 7b). However, the percentage of apoptotic immature MTS20^+^ cells was lower in mutant thymi at E17.5, but increased to reach significantly higher values than control WT ones in 1 M adult thymi (Fig. 7b). The proportions of apoptotic cTECs were significantly lower in 1 M mutant mice (Fig. 7b), in which the values of Ly51^+^ cells were higher in mutant than in WT mice (Fig. 4a). A similar correlation could be established between the apoptotic rate (Fig. 7b) and the proportions of UEA1^+^ mTECs at E17.5 (Fig. 4b).

### Thymocyte development in β-catenin-deficient thymi

**Fig. 8:**
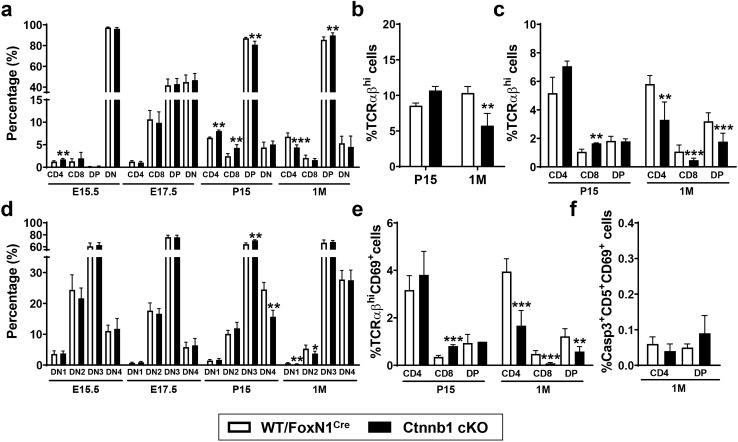
Analysis of the developing thymocyte subpopulations in the WT/FoxN1^Cre^ and Ctnnb1 cKO thymi. The proportions of WT and mutant thymocyte subsets [DN, CD4^−^CD8^−^; DP, CD4^+^CD8^+^; SP, CD4^+^CD8^−^ (CD4), CD4^−^CD8^+^ (CD8)] defined by CD4/CD8 expression are shown in panel **a**, whereas those showing total mature TCRαβ^hi^ cells and TCRαβ^hi^CD4^+^, TCRαβ^hi^CD8^+^ and TCRαβ^hi^DP cell subsets are in panel **b** and **c**, respectively. **d** Proportions of DN cell subsets: DN1 (CD44^+^CD25^−^), DN2 (CD44^+^CD25^+^), DN3 (CD44^−^CD25^+^) and DN4 (CD44^−^CD25^−^) were evaluated in the Lin^−^cKit^−/lo^ cell population. Frequency of positively (TCRαβ^hi^CD69^+^CD4^+^, TCRαβ^hi^CD69^+^CD8^+^ and TCRαβ^hi^CD69^+^DP) and negatively selected (Casp3^+^CD5^+^CD69^+^CD4^+^ and Casp3^+^CD5^+^CD69^+^DP) thymocytes are shown in panels **e** and **f**, respectively. The significance of the Student’s *t* test probability between WT and mutant is indicated as: *p ≤ 0.05, **p ≤ 0.01 and ***p ≤ 0.001

Then, we evaluated whether described phenotypical changes in the maturation of TEPCs, particularly those of the cortical TEC lineage, affected thymocyte differentiation. Because the numbers of lymphoid cells are very low in the earliest stages of thymus development, the maturation of distinct thymocyte subpopulations was evaluated from E15.5 onward (Fig. 8).

Distinct patterns of thymocyte maturation between WT and β-catenin-deficient TEC thymi were only evident after birth, especially in adult thymus, and largely affected the proportions of DP thymocytes (Fig. 8a) and mature TCRαβ^hi^ cells (Fig. 8b), suggesting a partial blockade of the final maturation of the DP cell compartment. Thus, some correlation appears to exist between the profile of thymocyte maturation in P15 and adult mutant mice and the above-described delayed maturation of thymic cortex. In 1 M adult thymi, increased proportions of DP thymocytes (Fig. 8a) and a significant decrease in all TCRαβ^hi^ cells (Fig. 8b), including DP, CD4 and CD8 thymocytes, were observed (Fig. 8c). When the proportions of positively (Fig. 8e) and negatively selected thymocytes (Fig. 8f) were examined in mutant and WT adult thymi, a lower percentage of positively selected TCRαβ^hi^CD69^+^ cell populations, particularly those either expressing CD4 or CD8, occurred in the mutant adult thymi (Fig. 8e), in correlation with the low proportions of mutant total TCRαβ^hi^ thymocytes (Fig. 8b). On the other hand, no changes in the negative selection were found and similar proportions of both mutant and WT Casp3^+^CD5^+^CD69^+^CD4^+^ cells and Casp3^+^CD5^+^CD69^+^DP cells (Fig. 8f) were found.

At P15, this apparent blockade of DP progression was not yet so evident and the proportions of mutant DP thymocytes were significantly lower than those of WT ones (Fig. 8a), in correlation with the increased percentages of TCRαβ^hi^ cells (Fig. 8b), largely corresponding to TCRαβ^hi^CD8^+^ cells (Fig. 8c).

No significant variations occurred in the proportions of total DN cells at any stage (Fig. 8a), but when possible changes in the proportions of DN1-DN4 cell subsets were examined, the proportions of both DN1 and DN2 cells were significantly reduced in 1 M mutant thymi (Fig. 8d). This would suggest that in the adult thymus, lower numbers of lymphoid progenitor cells could be maturing in the Ctnnb1 cKO thymi. At P15, the proportions of DN4 cells declined significantly, owing to a build-up of DN3 cells (Fig. 8d), a finding that could contribute to the low proportions of DP thymocytes in mutant thymi reported here (Fig. 8a).

### Peripheral lymphoid cell populations in β-catenin-deficient mice

**Fig. 9:**
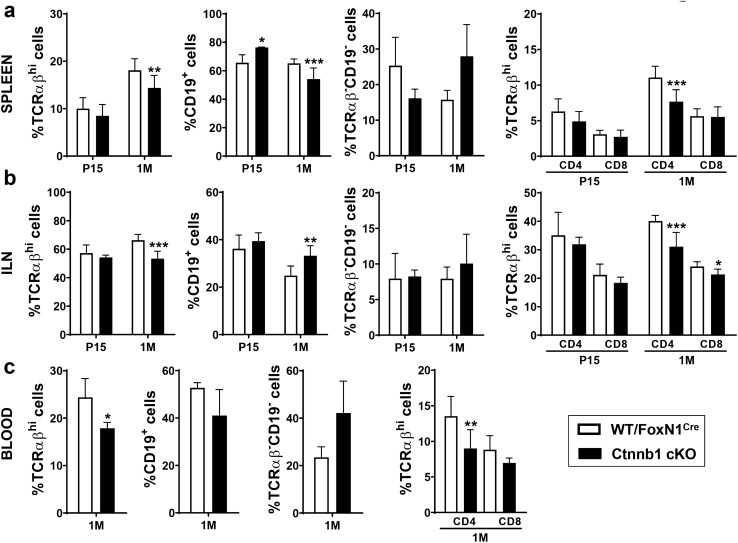
Analysis of the proportions of T- and B-lymphocytes in peripheral lymphoid tissues of WT/FoxN1^Cre^ and Ctnnb1 cKO mice. The proportions of control and mutant peripheral lymphocytes, including total TCRαβ^hi^, CD19^+^, TCRαβ^−^CD19^−^, TCRαβ^hi^CD4^+^ and TCRαβ^hi^CD8^+^cells, are shown in spleen (**a**) and inguinal lymph nodes (ILNs) (**b**) at P15 and 1 M, and at 1 M in peripheral blood (**c**). The significance of the Student’s *t* test probability between WT and mutant is indicated as: *p ≤ 0.05, **p ≤ 0.01 and ***p ≤ 0.001

Because altered levels of β-catenin in the thymus have been correlated with changes in peripheral lymphoid subpopulations (Ma et al. 2012), we evaluated how this misfunction of mutant TECs impacted on the peripheral lymphoid organs. We analysed the condition of the spleen, ILN and peripheral blood in postnatal and adult mutant and WT mice (Fig. 9). In the three tissues, variations between mutant and WT values were restricted to 1 M adult mice and consisted of significantly reduced proportions of TCRαβ^hi^-expressing T cells (Fig. 9a, b, c). The proportion of CD19^+^ B lymphocytes was also lower in both spleen and peripheral blood (Fig. 9a, c), but increased in the ILNs (Fig. 9b). These reductions correlated with a non-significant increase in the TCRαβ^−^CD19^−^ non-T/non-B cells (Fig. 9a, c). Within the T cell population, the reduced proportions of TCRαβ^hi^ cells largely corresponded to CD4^+^ cells in the three tissues studied, but in the case of the ILNs CD8^+^ T cells also contributed (Fig. 9b).

### Transcript levels of some molecules related to Wnt signalling vary in β-catenin-deficient thymi

**Fig. 10:**
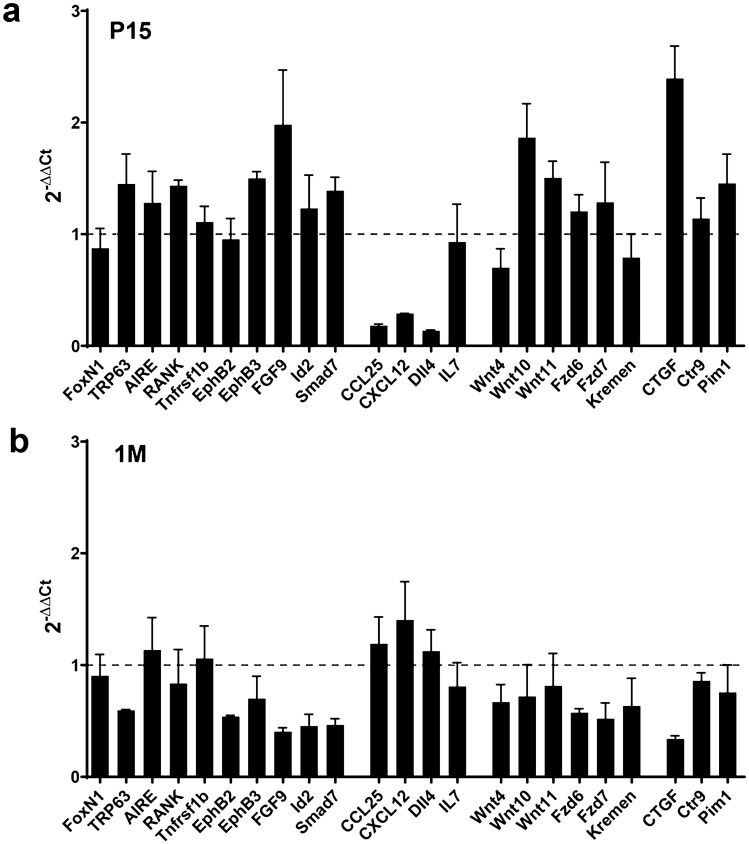
Analysis by qPCR of the expression of different genes known to be associated with TEC development in Ctnnb1 cKO thymi compared with that observed in WT/FoxN1^Cre^. The figures show the relative expression (2^−ΔΔCt^) of different genes in Ctnnb1 cKO EpCAM^+^CD45^−^TECs (black bars) at P15 (**a**) and 1 M (**b**) with respect to WT/FoxN1^Cre^ TEC values (value = 1; dashed line)

A first glance at our qPCR results indicated that, in general terms, the differences in the transcript values in mutant and WT thymi were more evident at 1 M, presumably reflecting the more severe phenotype found in the 1 M adult mutant thymi than in the P15 ones (Fig. 10).

As indicated above, previous investigations have demonstrated that Wnt signalling regulates thymic epithelial maturation (Balciunaite et al. 2002; Liang et al. 2013), and the expression of molecules involved in this signalling pathway, including ligands and receptors, are governed differentially in distinct cell types and throughout thymus growth (Pongracz et al. 2003; Heinonen et al. 2011; Varecza et al. 2011; Kvell et al. 2014). Therefore, we analysed four categories of molecules (Fig. 10):

- Those involved in the maturation of thymic epithelium: FoxN1, TRP63, AIRE, RANK, Tnfrsf1b, EphB2, EphB3, FGF9, Id2 and Smad7, or
- Those involved in its activity: CCL25, CXCL12, Dll4 and IL7;
- Ligands, receptors and molecules involved in the signalling of Wnt pathways: Wnt4, Wnt10, Wnt11, Fzd6, Fzd7 and Kremen;
- Others: CTGF, Ctr9, Pim.

Among the molecules known to govern thymic epithelial development, our results did not find substantial differences in the transcript numbers of FoxN1 and Tnfrsf1b between mutant and WT EpCAM^+^ cells. The values were also quite similar in P15 and 1 M thymi (Fig. 10). Contrarily, other molecules included in this group showed a remarkable profile, exhibiting higher values in P15 mutants (Fig. 10a) but a robust, significant decrease in 1 M mutant thymi versus WT values (Fig. 10b). This group included TRP63, RANK, EphB2, EphB3, FGF9, Id2 and Smad7. Other molecules, such as AIRE, exhibited slight changes at both P15 and 1 M (Fig. 10a, b). The values of some transcripts, such as those of chemokines CCL25, CXCL12 and Dll4, produced by TECs but affecting the migration and differentiation of thymocytes, changed significantly between P15 and 1 M (Fig. 10). In the first stage, mutant values were significantly lower than those of WT ones, but in 1 M mutant levels were higher than WT ones. In this same group, IL7 transcripts did not undergo remarkable changes (Fig. 10).

On the other hand, the transcript numbers of molecules associated with the Wnt signalling pathway decreased in 1 M mutant thymi, particularly in the case of Wnt10, Wnt11, Fzd6 and Fzd7, which showed increased values compared with WT ones in P15 TECs (Fig. 10a), but a significant reduction at 1 M (Fig. 10b). Finally, the important fall in mutant values of other molecules associated with the Wnt signalling pathway, such as Ctr9 and Pim1, was noteworthy and especially CTGF (Connective Tissue Growth Factor) (Fig. 10b). This latter molecule is a member of the family of connective matrix-associated heparin-binding proteins involved in tissue healing and fibrotic disease (Hall-Glenn and Lyons 2011), which is part of a negative feedback loop of the β-catenin-dependent Wnt signalling pathway (Luo et al. 2004; Mercurio et al. 2004; Varecza et al. 2011).

## Discussion

Our current results demonstrate that the specific deletion of β-catenin in FoxN1^+^ TECs corresponds to profound hypocellularity, delay and partial blockade of the maturation of thymic epithelium, largely affecting immature MTS20^+^ cells, presumptive bipotent Ly51^+^MTS20^+^MHCII^hi^ adult TEPCs and cTECs. This abnormal maturation of thymic epithelium after the selective abrogation of β-catenin in TECs also affects T-cell differentiation that corresponds to significant alterations especially in postnatal and adult thymi. On the other hand, peripheral T and B lymphocytes of spleen, ILNs and peripheral blood also appear to be affected in mutant adult mice. Together, these results confirm that TECs are the main cell targets for the action of β-catenin-mediated canonical Wnt signalling pathway in the thymus, although direct effects on thymocytes have also been reported (Ma et al. 2012).

Several studies have proposed a role for β-catenin-mediated Wnt signalling in the development and homeostasis of TECs (Zuklys et al. 2009; Liang et al. 2013; Bredenkamp et al. 2014), but others claim that a low or null Wnt signalling is mandatory for thymus development (Swann et al. 2017). In part, these contradictory results reflect different experimental approaches and/or developmental stages studied, suggesting that the role of β-catenin in the thymus might vary in different cell types and developmental stages. A survey of the literature devoted to the role of Wnt signalling pathway in the thymus shows that the abrogation of Wnt signalling, via different pathways, corresponds to an overall delay of TEC maturation (Kuraguchi et al. 2006; Osada et al. 2010; Liang et al. 2013; Kvell et al. 2014; Brunk et al. 2015). However, activation of the Wnt pathway, by either disruption of inhibitors of the Wnt pathway or constitutive expression of active β-catenin, also produces severe epithelial failure with strong keratinization, terminal TEC differentiation or thymoma development (Osada et al. 2006; Zuklys et al. 2009; Kvell et al. 2014; Liang et al. 2015; Swann et al. 2017). Nevertheless, this regressive epithelial maturation could also be associated with the massive production of Wnt inhibitors that was reported following expression of stabilized forms of β-catenin (Mulroy et al. 2002; Niida et al. 2004).

Mice exhibiting selective abrogation of β-catenin in FoxN1^+^ cells, such as those examined in this work, were previously studied by Swann and colleagues (2017). In agreement with our current results, they also observed severe hypocellularity in foetal mutant thymi, but only a slight decrease in the proportions of Ly51^+^ cells at E15, but not of CD80^+^ cells and UEA1^+^ cells. Our current study extends these results, concluding that this low number of TECs was related to an accumulation of MTS20^+^Ly51^+^ cells. This previous report only referred to embryonic thymi because mice died after birth because of skin problems. This was due to the absence of β-catenin also affecting the basal epidermal cells that express FoxN1 (Swann et al. 2017). Although > 50% of newborn mutants also died in our study, those that did survive showed a more severe thymic phenotype than that observed in embryonic mice. In correlation, β-catenin expression by TEC in WT mice significantly increases after birth, especially in 1 M, adult thymi, which is when our study finds the most important changes in mutant thymi.

Our results show that after birth, the mutant MTS20^+^Ly51^+^ cell population, whose percentage increases during development, is accumulated. In addition, both MTS20^+^Ly51^+^MHCII^hi^ and MTS20^+^Ly51^+^MHCII^lo^ cTEC subsets also accumulate importantly in mutant mice. Although TEPCs are assumed to exist in adult thymus, their phenotype is a matter of discussion. Blackburn and colleagues identified bipotent TEPCs expressing Plet1, a cell marker recognized by mAbs MTS24 and MTS20, different from a major medullary Plet1^+^ cell population, which expressed both the cortical cell marker Ly51 and MHCII^hi^ molecules, while Ly51^+^Plet1^+^(MTS20^+^)MHCII^lo^ cells presented mainly cTEC progenitor-restricted potential (Ulyanchenko et al. 2016). Therefore, our current results indicate that in the adult thymus these cell types, Ly51^+^MTS20^+^MHCII^lo^ population containing cTEC restricted progenitors and Ly51^+^MTS20^+^MHCII^hi^ containing potentially bipotent progenitors, rather than corresponding to more mature Ly51^+^ cTECs and UEA1^+^ mTECs, would, in fact, be the cellular targets of the Wnt signalling pathway.

Most published studies have analysed changes in the proportions of cTECs, mTECs and TEPCs in specific stages of development, mainly in adult thymi. However, there is little information on changes in these cell subsets throughout thymus development, making comparisons with our results difficult. Some studies report reduced numbers of both cTECs and mTECs (Osada et al. 2010), but more frequently a decline in the proportions of mTECs are observed (Heinonen et al. 2011; Liang et al. 2013) with increased proportions of cTECs (Liang et al. 2013), as described herein, or no changes in the cortical epithelium (Heinonen et al. 2011). On the contrary, experimental protocols performed to maintain the Wnt signalling pathway constitutively active drive the thymic epithelium to exhaustion with a dramatic reduction in all TEC subsets, altered thymic histology, increased K8^+^K5^+^ cells and epithelial-free areas (EFAs) (Osada et al. 2006; Zuklys et al. 2009). Indeed, these results are not controversial if, as mentioned above, we consider that the lack or overexpression of Wnt signalling in different thymic cell types could result in distinct phenotypes. Furthermore, the Wnt pathway is quite autoregulated and expression of different members affects the expression of others (Mulroy et al. 2002; Niida et al. 2004), as our qPCR results also demonstrated. Furthermore, the strength and/or activation timing of Wnt signalling could determine different TEC outcomes, in some cases partially blocking TEC maturation and inducing their differentiation to a final stage of severe squamous keratinized epithelium in others (Liang et al. 2015; Swann et al. 2017). In this regard, one feature that could possibly explain these opposite results is the different expression of β-catenin. This could be a way to check the activity of Wnt signalling (Staal and Langerak 2008; Clevers and Nusse 2012) by distinct TEC subsets in WT thymi. Thus, the most severe effects observed in both Ly51^+^ cTECs and immature MTS20^+^ cells correlate well with the levels of β-catenin observed in WT TEC subsets and their evolution throughout thymus development, a finding also reported by other authors (Liang et al. 2013).

In general, the absence of Wnt signalling, due to inhibitors of the canonical Wnt pathway (Osada et al. 2010; Kvell et al. 2014), absence of Wnt ligands (Heinonen et al. 2011; Brunk et al. 2015) or deletion of β-catenin in K5-expressing TECs (Liang et al. 2013), corresponds to hypocellularity that can eventually result in premature thymic degeneration (Osada et al. 2010). There is controversy regarding the origin of this low thymic cell content. Although most studies report reduced total TEC numbers, as discussed above, the TEC subsets involved are a matter of discussion. Remarkably, in most studies the rule is that there are no changes in epithelial cell proliferation (Heinonen et al. 2011; Liang et al. 2013; Brunk et al. 2015), although decreased TEC survival has been reported occasionally (Brunk et al. 2015). Our results also demonstrate few changes in the proportions of cycling and apoptotic total EpCAM^+^ cells in the mutant thymi, making it difficult to explain the changes observed in the percentages of mature cTEC and mTECs. Indeed, when the proportions of both cycling and apoptotic cells in the distinct TEC subsets are analysed, the TEC population most affected corresponds to immature MTS20^+^ cells, a finding that could affect their maturation.

Overall, rather than indicating a relevant role for β-catenin signalling in TEC proliferation or survival, our results would reveal its importance in the differentiation of TECs, both cortical and mTEC from bipotential progenitors, and also of mature cTECs from immature cTEC-restricted progenitors, to some extent throughout development, but mainly in the postnatal thymus. A general role of Wnt pathway in regulating progenitor maintenance and differentiation, especially in adult tissues, has been extensively described (Liang et al. 2013; Swann et al. 2017; Tan and Nusse 2017). Therefore, mutant Ctnnb1 cKO thymi would have problems for the transition from common TE precursor cells to both cTECs and mTECs, as well as an altered development of mature cTECs from cortical TEC-restricted progenitors. Contrarily, the absence of β-catenin signalling does not seem to affect the maturation of restricted mTEC precursor cells.

On the other hand, our qPCR analysis of the transcript expression of diverse molecules related to TECs could explain some of the changes observed. As is known, FoxN1 is an essential transcription factor for proper thymic epithelial development; mutant EpCAM^+^ TECs show slightly reduced FoxN1 transcripts, but other molecules acting downstream of FoxN1, also involved in TEC maturation, significantly decrease at 1 M, including EphB3 involved in cortical epithelial maturation (Montero-Herradon et al. 2017) or both RANK and EphB2 implicated in mTEC development (Montero-Herradon et al. 2018).

Together with these alterations in TECs, the thymi of mice exhibiting a specific deletion of β-catenin in FoxN1^+^ TECs show changes in the pattern of T-cell differentiation. Overall, as reported for TEC maturation, mutant thymi show delayed maturation of thymocytes particularly in 1 M mice. Although some studies have reported no changes in thymocyte maturation in thymi with β-catenin-mediated altered TECs (Osada et al. 2006, 2010), or these are limited to the earliest stages of T-cell development (i.e., DN1 and DN2 cells) (Heinonen et al. 2011), in general, altered Wnt signalling (absence or overexpression) in TECs corresponds to concomitant changes in T-cell differentiation, presumably as a consequence of the altered TEC development. In the current study, we observed decreased proportions of DN1 cells and delayed maturation of the DP cell compartment, that first (P15) corresponds to reduced proportions of DP cells and later (1 M) to their accumulation. Moreover, in mice exhibiting sustained canonical Wnt signalling in which the thymic epithelial microenvironment is severely involuted, a total blockade of T-cell differentiation occurs (Zuklys et al. 2009; Swann et al. 2017). On the other hand, it is assumed that the main source of Wnt ligands in thymus is the TECs (Pongracz et al. 2003; Brunk et al. 2015) and that their absence from the thymic epithelium is not supplied by their expression on thymocytes (Brunk et al. 2015).

Transcripts of both ligands and receptors of Wnt signalling pathway significantly reduce the main ligand of the canonical Wnt pathway (Pongracz et al. 2003), particularly Wnt10 in 1 M mutant TECs. Therefore, it is possible to speculate that in these conditions β-catenin defective TECs would only poorly activate the Wnt receptor expressing thymocytes. In fact, many features found in the T-cell differentiation of our mutants, such as thymic hypocellularity, partially impeded maturation of DN to DP and then to mature SP thymocytes, and reduced peripheral T lymphocytes, have been described in experimental models which lack Wnt ligand-expressing cells (Brunk et al. 2015). Other authors have reported that the absence of Wnt signalling compromises the maturation of DN cells (Verbeek et al. 1995) through preTCR-mediated T-cell selection (Gounari et al. 2001, 2005).

On the other hand, 1 M mutant thymi exhibit decreased proportions of TCRαβ^hi^ cells and of positively selected TCRαβ^hi^DP cells, CD4 cells and CD8 cells, but there are no changes in the percentages of negatively selected thymocytes, although our semiquantitative histochemical and qPCR analysis indicate a slight increase in AIRE expression in mutant thymi. This defective maturation of thymocytes and the reduced proportions of positively selected T cells could be related to the altered development of thymic cortical epithelium reported herein, a finding also described in conditioned mice in which TECs are unable to secrete Wnt ligands (Brunk et al. 2015). In addition, subtle changes in β-catenin signalling in DP thymocytes could affect the selection outcome, as other authors have proposed (Ma et al. 2012). On the other hand, the partial blockade of DN cell progression to the DP cell stage correlates with reduced expression in the P15 mutant EpCAM^+^ TECs of some molecules, such as CCL25, CXCL12 and Dll4, implicated in the maturation of DP thymocytes (Lancaster et al. 2018). Later, their expression increases in correlation with the increased proportions of DP cells.

In addition, the intrathymic behaviour of developing thymocytes of mutant mice is also reflected in the peripheral T-cell populations. Hence, proportions of TCRαβ^hi^ T cells decreased in all three tissues studied here. Moreover, in both spleen and peripheral blood the reduced TCRαβ^hi^ T cells largely correspond to CD4^+^ cells whereas in ILNs the TCRαβ^hi^ cell reduction correlates well with declines in populations of both CD4^+^ and CD8^+^ cells. There are few data on the behaviour of peripheral lymphoid cell subsets in mice with selective abrogated β-catenin in TECs. In agreement with our findings, Brunk et al. (2015) reported that the lack of TEC provided Wnt ligands leading to a reduced peripheral T cell pool but, in contrast to our results, the proportions of specific T cell subsets did not change and peripheral T lymphocytes were functionally competent.

Taken together, the current results confirm an altered maturation of thymic epithelium, largely affecting immature/progenitor TECs in mice with the β-catenin gene selectively deleted in FoxN1^+^ TECs. This altered thymic microenvironment also affects T-cell differentiation, particularly of the DP cell compartment, and correlates with changes in the expression of various molecules that act downstream of Wnt signalling governing TEC differentiation.

## Supplementary Information

Below is the link to the electronic supplementary material.

Supplementary file1 (DOCX 702 KB)

## Data Availability

The datasets generated during and/or analysed during the current study are available from the corresponding author on reasonable request.
