# Supplementary material for: Delayed maturation of thymic epithelium in mice with specific deletion of β-catenin gene in FoxN1 positive cells
Source: Histochem Cell Biol. 2021 Jul 12;156(4):315–32. doi: 10.1007/s00418-021-02012-w (PMC8550644; doi:10.1007/s00418-021-02012-w)
Supplement: Supplementary file 1 — Supplementary file1 (DOCX 702 KB) [file 418_2021_2012_MOESM1_ESM.docx]

**Delayed maturation of thymic epithelium in mice with specific deletion of β-catenin gene in FoxN1 positive cells**

***Sara Montero-Herradón****^1^* ***and Agustín G. Zapata****^1†^*

^1^ Department of Cell Biology, Faculty of Biology, Complutense University of Madrid, 28040, Madrid, and Health Research Institute, Hospital 12 de Octubre (imas12), Madrid, Spain.

^†^Corresponding author: Dr. Agustín G. Zapata, Department of Cell Biology, Faculty of Biology, Complutense University of Madrid, C/ José Antonio Nováis 2; 28040 Madrid, Spain; Phone: +34 91 394 4979; Fax: +34 91 394 4981; e-mail address: zapata@ucm.es

ORCID: Montero-Herradón S: 0000-0003-2004-8987; Zapata AG: 0000-0003-0576-2672

| **Primer** | **Primer Type** | **Sequence 5' 🡪 3'** |
| --- | --- | --- |
| 15344 | FoxN1 Wild type *Forward* | CCT ATG CCA CTC AGC CAA CT |
| 15345 | FoxN1 Wild type *Reverse* | GGA GGG GTG ATC TTT GAC CT |
| oIMR1084 | FoxN1 Mutant *Forward* | GCG GTC TGG CAG TAA AAA CTA TC |
| oIMR1085 | FoxN1 Mutant *Reverse* | GTG AAA CAG CAT TGC TGT CAC TT |
| oIMR1512=RM41 | β-catenin *Forward* | AAG GTA GAG TGA TGA AAG TTG TT |
| oIMR1513=RM42 | β-catenin *Reverse* | CAC CAT GTC CTC TGT CTA TTC |
| RM43 | β-catenin *floxdel* | TAC ACT ATT GAA TCA CAG GGA CTT |

**Online Resource 1**. Primers used for mouse genotyping.

| **Antibody** | **Fluorochrome** | **Clone** | **Company** |
| --- | --- | --- | --- |
| anti-CD4 | PerCP, APC | GK1.5 | Biolegend |
| anti-CD8α | APC, PE, Pacific Blue | 53-6.7 | Biolegend |
| anti-TCRβ | AlexaFluor488 | H57-597 | Biolegend |
| anti-CD44 | FITC | IM7 | Biolegend |
| anti-CD117 (cKit) | PE | 2B8 | Biolegend |
| anti-CD25 | PerCP | PC61 | Biolegend |
| anti-Lineage (Lin) cocktail | APC | --- | BD Biosciences |
| anti-CD5 | FITC | 53-7.3 | Biolegend |
| anti-CD69 | APC | H1.2F3 | Biolegend |
| anti-Caspase3 | PE | 5A1E | Cell Signalling Technology |
| anti-EpCAM | PE, APCCy7 | G8.8 | Biolegend |
| anti-CD45 | APCCy7, Pacific Blue, AlexaFluor647 | 30-F11 | Biolegend |
| anti-Ly51 | AlexaFluor647 | 6C3 | Biolegend |
| anti-MHCII | Pacific Blue | M5/114.15.2 | Biolegend |
| anti-CD80 | BrilliantViolet605 | 16-10A1 | Biolegend |
| anti-β-Catenin | AlexaFluor488 | 14 | BD Biosciences |

**Online Resource 2.** Antibodies used for the flow cytometric analysis.

| **Gene** | **Reference** |
| --- | --- |
| FoxN1 | Mm00433948_m1 |
| Dll4 | Mm00444619_m1 |
| Fzd6 | Mm00433387_m1 |
| Fzd7 | Mm00433409_s1 |
| Wnt10a | Mm00437325_m1 |
| Wnt4 | Mm01194003_m1 |
| Wnt11 | Mm00437328_m1 |
| Kremen | Mm00459616_m1 |
| TRP63 | Mm00495793_m1 |
| AIRE | Mm00477461_m1 |
| Tnfrsf11a (RANK) | Mm00437132_m1 |
| IL7 | Mm01295803_m1 |
| Cxcl12 | Mm00445553_m1 |
| CCL25 | Mm00436443_m1 |
| EphB2 | Mm01181021_m1 |
| EphB3 | Mm00802553_m1 |
| CTGF | Mm01192933_g1 |
| FGF9 | Mm00442795_m1 |
| Tnfrsf1b | Mm00441889_m1 |
| Id2 | Mm00711781_m1 |
| Smad7 | Mm00484742_m1 |
| Ctr9 | Mm00493862_m1 |
| Pim1 | Mm00435712_m1 |
| HPRT | Mm00446968_m1 |

**Online Resource 3**. Taqman primers used in qPCR studies.


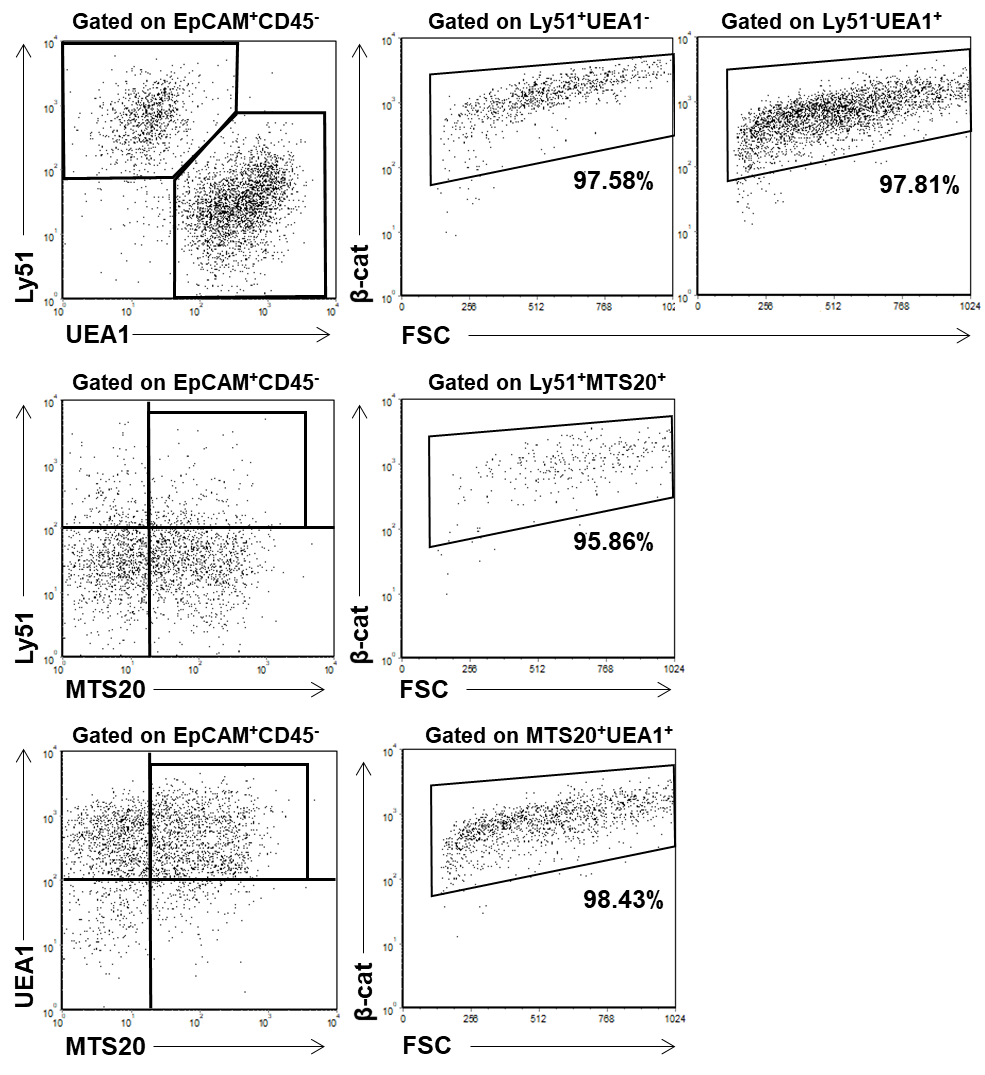


**Online Resource 4**. Representative FACS plots of the gating profile of different TEC subpopulations examined for β-catenin expression. The proportions of β-catenin expressing TECs are indicated.


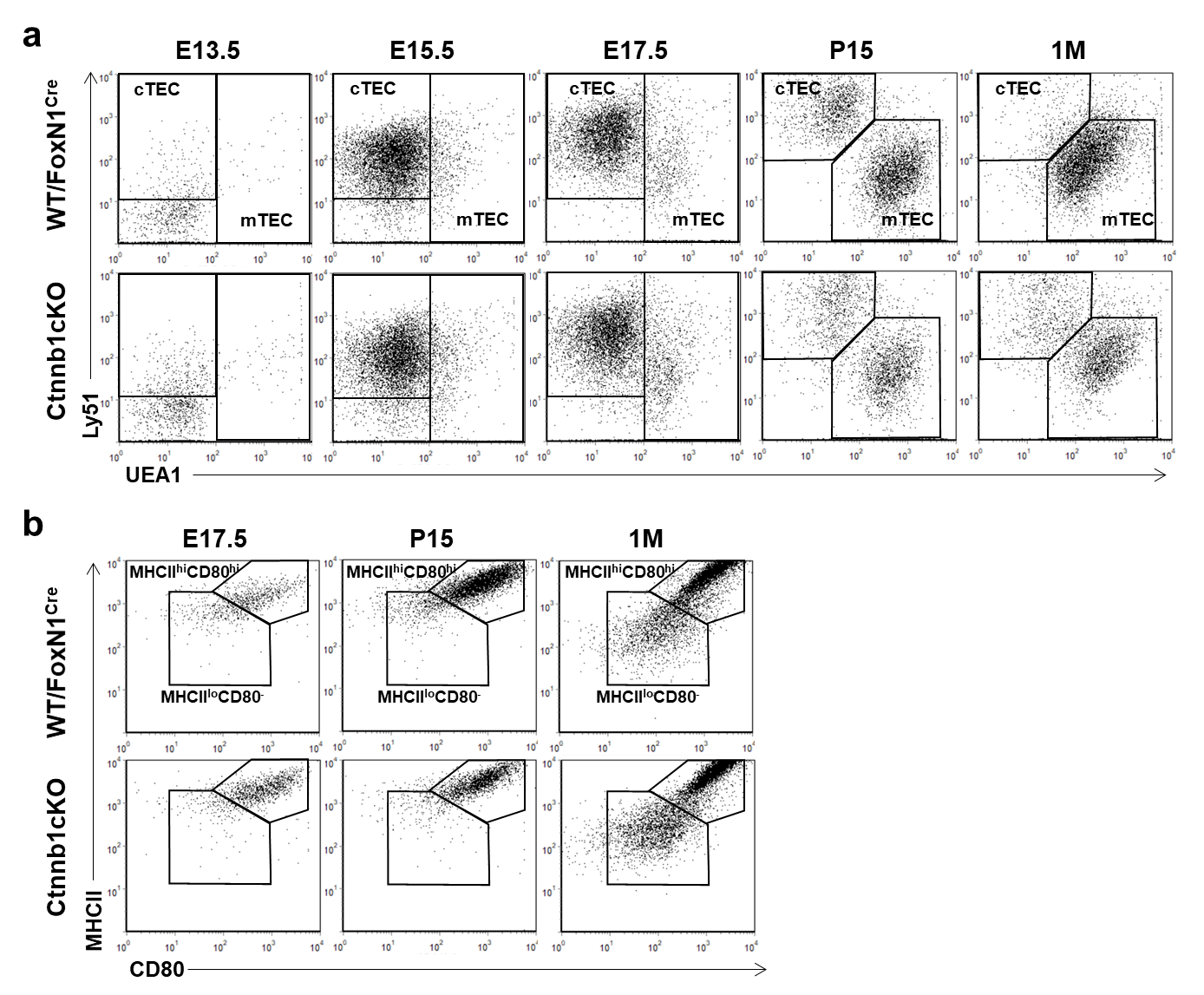


**Online Resource 5**. Representative FACS plots of the TEC subsets defined by **(a)** Ly51/UEA1 expression. **(b)** The figure shows the MHCII/CD80 expressing mTECs within the EpCAM^+^CD45^-^Ly51^-^UEA1^+^ cell population.
